# A randomized controlled trial of an interactive digital therapeutic for stress and burnout management

**DOI:** 10.1038/s44184-025-00184-0

**Published:** 2025-12-30

**Authors:** Katharina M. Rischer, Linda T. Betz, Antje Riepenhausen, Björn Meyer, Gitta A. Jacob, Helge Frieling, Kamila Jauch-Chara

**Affiliations:** 1 Research Department, GAIA, Hamburg, Germany; 2 Department of Psychiatry, Social Psychiatry and Psychotherapy, Hannover Medical School, Hannover, Germany and AWO Psychiatriezentrum Königslutter, Königslutter, Germany; 3 Department of Psychiatry and Psychotherapy, Christian-Albrechts-Universität zu Kiel, Kiel, Germany; 4 Vincera Klinik Bad Waldsee GmbH, Private Clinic for Psychosomatic Medicine und Psychotherapy, Bad Waldsee, Germany

**Keywords:** Psychology, Occupational health, Therapeutics

## Abstract

This pragmatic randomized controlled trial examined the effectiveness of *reviga*, a self-guided digital intervention based on cognitive behavioral therapy, in reducing work-related stress symptoms. A total of 290 adults experiencing significant stress and burnout were assigned to the intervention group (*reviga* + treatment as usual [TAU]; n = 147) or the control group (TAU only; n = 143). Intent-to-treat analyses showed that 3 months post-randomization, participants in the intervention group experienced significant positive effects on the primary outcome, perceived stress (Cohen’s *d* = 0.36), as well as on the secondary outcomes anxiety (*d* = 0.28), burnout (*d* = 0.31), occupational and social functioning (*d* = 0.31) and health-related quality of life (*d* = 0.35) compared to TAU. No effect was found for absenteeism quantified as the number of sick days. Effect sizes increased at 6 month follow-up. This study demonstrates that *reviga* represents a promising and scalable tool for workplace mental health support.

## Introduction

Work-related stress is a prevalent phenomenon across occupational sectors. A nationwide, representative survey by a major health insurer revealed that work is the top stressor in Germany, with 47% of respondents citing it as a source of stress^1^. At the same time, many employees feel that their employers do not provide adequate resources for managing stress^2^.

While short-term stress can potentially enhance task performance by facilitating adaptive responses to workplace demands, sustained exposure to stress has been associated with adverse effects on both physical and mental health^3,4^. Chronic workplace stress is a significant risk factor for the development of common mental health disorders, including depression and anxiety, particularly in individuals experiencing effort-reward imbalances^5^. Additionally, work-related stress has been implicated in sleep disturbances^6,7^ and broader societal consequences, such as increased absenteeism^8,9^, occupational disability^10^, and constitutes a risk factor for physical diseases^11^, contributing to a substantial public health burden. Given these wide-ranging implications, early intervention is essential to mitigate work-related stress and prevent burnout. As a key precursor to burnout, prolonged stress can lead to emotional, physical, and mental exhaustion, ultimately reducing professional efficacy and increasing detachment from work.

While traditional, face-to-face stress management interventions have demonstrated efficacy in reducing perceived stress levels^12–14^, they are not readily available due to scarcity and long wait times for in-person resources, especially psychotherapy^15,16^. At the same time, many patients are open to digital self-management interventions^17^. However, 60% of those who self-manage express dissatisfaction with the outcomes of their efforts^2^. Although numerous digital solutions for stress management are available, the vast majority lack an evidence base^18^. Of those that are evidence-based, many are narrow in scope, typically focusing on specific techniques such as breathing exercises or relaxation techniques^18,19^. This highlights a significant gap and underscores the urgent need for more accessible, evidence-based digital interventions that can provide timely, scalable support for work-related stress. A recent meta-analysis of 21 randomized controlled trials (RCTs) on web-based psychological interventions in the workplace found significant reductions in perceived stress, depression, and psychological distress (*d* = 0.37, 95% CI 0.23–0.50)^20^. These findings demonstrate the potential of digital interventions to alleviate work-related psychological strain effectively.

*reviga* is a self-guided, digital intervention grounded in cognitive-behavioral therapy (CBT), specifically designed for individuals experiencing stress and burnout. As a flexible, resource-efficient solution, *reviga* requires no professional oversight and is accessible via the Internet at the user’s convenience. The program delivers personalized psychoeducation and therapeutic exercises tailored to individual needs and preferences. Features such as personalized content, automated reminders, and progress tracking are incorporated to promote consistent engagement and motivation.

This randomized controlled trial (RCT) evaluated the efficacy of *reviga* in alleviating perceived stress and burnout among adults experiencing work-related stress. The study included a Treatment as Usual (TAU) control group, which reflects the standard care typically available to individuals, including both informal workplace support programs and access to professional resources such as counseling or therapy. By evaluating the effectiveness of *reviga* within a diverse sample, this trial aimed to examine its utility for improving workplace mental health.

## Methods

### Recruitment and assessment

This study was reviewed and approved by the ethics committee of the medical faculty at the Christian-Albrechts-Universität zu Kiel (reference number D 574/22). Prior to recruitment, the study “Evaluating the Effectiveness of a Digital Therapeutic (*reviga*) for people with stress and burnout - a randomized controlled trial (LAVENDER)” was registered in an international study register (NCT05998161). German-speaking participants were recruited through an online advertising campaign, primarily using targeted Google Ads to attract individuals experiencing stress and burnout. This approach was designed to efficiently reach the intended audience, directing interested individuals to a dedicated study website where they could find detailed information about the study and express their interest in participating. After electronically providing informed consent to participate in the study, participants completed an initial online survey. Following verification of the inclusion criteria, participants were randomly assigned to either the intervention or control group using an automated randomization system. This system implemented simple randomization, mimicking a digital coin toss that was automatically triggered for each participant immediately upon their inclusion. Technically, this was achieved using a custom Python script executing the *random.sample* function from Python’s standard *random* library to select either ‘intervention’ or ‘control’. This process ensured a 1:1 allocation ratio (without blocked randomization or stratification). Allocation concealment was maintained as the automated, real-time assignment could not be influenced or predicted by the research team. Due to the nature of the two study groups, participants were not blinded to their group assignment.

Throughout the trial, participants in the intervention group were provided access to *reviga* in addition to TAU while those in the control group received only TAU and were given access to *reviga* once they completed the study. TAU allowed participants in both groups to continue or discontinue any treatments, such as psychotherapy or medication. Participants in both groups were asked to complete online assessments at 3 months (T1; primary assessment point for evaluating effectiveness) and again at 6 months (T2; follow-up). All study procedures were conducted online.

### Inclusion and exclusion criteria

Participants were eligible for inclusion if they were between 18 and 65 years of age, experienced above-average perceived stress levels (PSS-10 score > 21) and burnout (OLBI score ≥ 2.18). Additionally, they needed to be residing in Germany, be employed for at least 20 h per week, and have maintained a stable treatment regimen for at least 30 days prior to enrollment. Participants were also required to provide informed consent to take part in the study. Individuals were excluded if they had plans to change their treatment within the next 3 months at the time of inclusion. Changes in treatment were defined as the start or end of psychotherapy or changes in medication received or taken by participants due to their stress and burnout symptomatology.

### Intervention

*reviga* is an internet-based, individually-tailored digital health application intended to support adults (18 years and older) in the self-management of stress and burnout. It is designed for standalone use as an adjunct to usual care and is not intended to replace treatment from a healthcare provider or to provide diagnostic information.

**Fig. 1:**
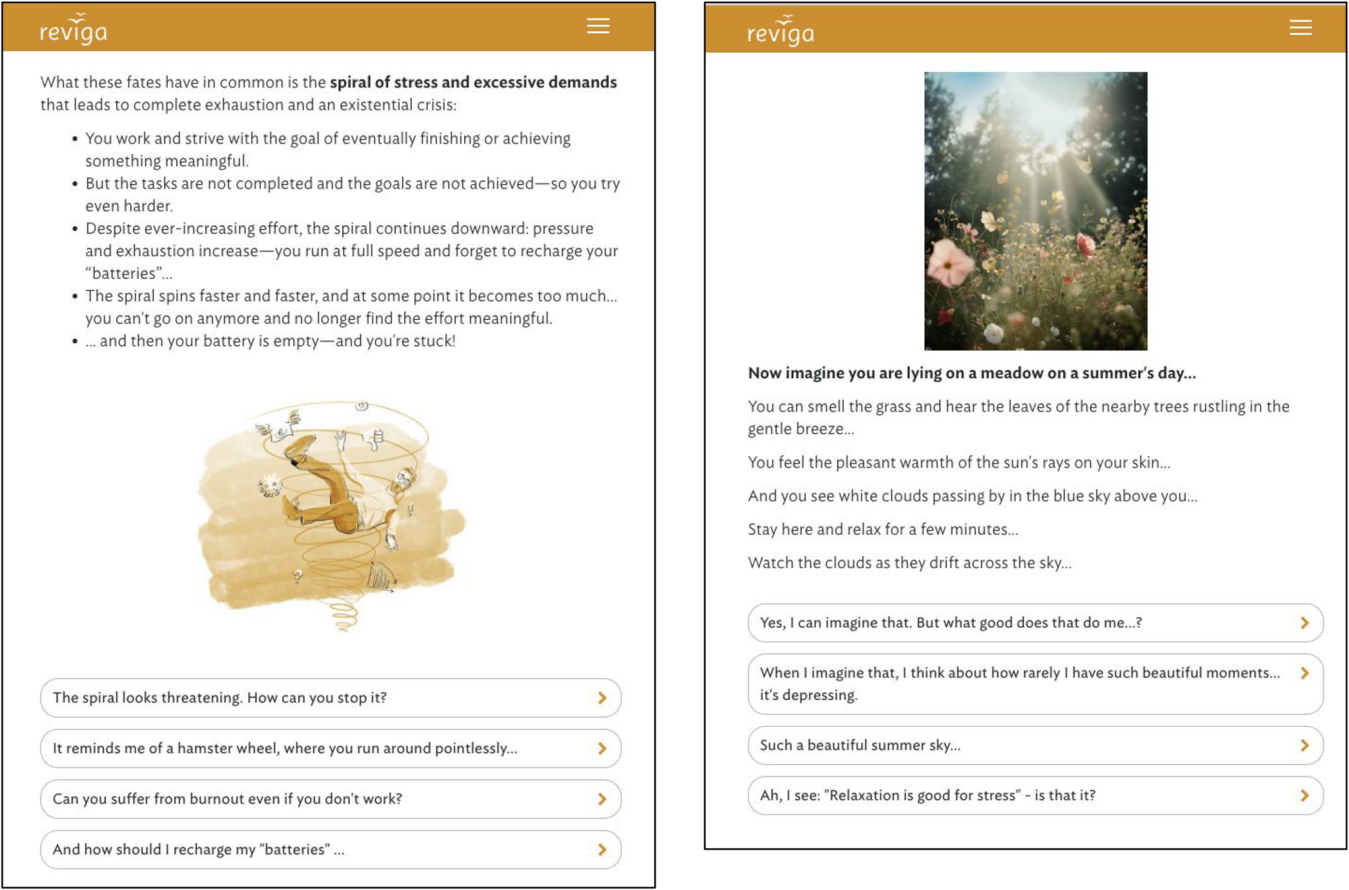
Selected screenshots of *reviga’s* content. *Note:* Translated to English for illustrative purposes. The study was conducted in German. An English version of *reviga* is currently not available.

The program was developed by a multidisciplinary team and is built on the proprietary *broca* software platform, which uses rule-based algorithms to mimic a dialog-like experience. Users interact by reading brief text passages and selecting from predefined response options, which allows the program to tailor subsequent content and create an individualized therapeutic dialog, a technique successfully used previously^21–24^. Screenshots of the program’s content are shown in Fig. 1.

The therapeutic content is evidence-based, integrating strategies from Cognitive-Behavioral Therapy (CBT), Acceptance and Commitment Therapy (ACT), and positive psychology. This content is structured into nine therapeutic topics (Table 1) and delivered via interactive text, illustrations, audio recordings (e.g., relaxation exercises), and downloadable PDF worksheets. To support engagement, the program provides optional motivational messages via email or SMS. No contraindications are noted.

**Table 1:** Content of *reviga*

Understanding Stress and Burnout	• Overview of stress and burnout, their causes, and effects on well-being • Explanation of treatment approaches and coping mechanisms • Self-assessment of stress symptoms • Introduction to relaxation techniques
Relaxation	• Strategies to cope with stress through physical activity and nutrition • Motivation for lifestyle changes to improve resilience • Training in relaxation and mindfulness techniques to reduce stress levels
Cognition	• Understanding the connection between thoughts, emotions, and stress • Recognizing automatic thoughts and creating distance from them • Identifying and addressing common thought traps (e.g., rumination, avoidance, catastrophizing) • Training in reframing and developing healthier thought patterns
Healthy Habits and Activities	• The role of basic needs (e.g., sleep, nutrition, exercise) in stress management • Encouraging positive and meaningful activities to counteract stress and burnout • Developing a personalized activity plan for well-being
Acceptance	• Identifying and accepting negative thoughts and emotions without being overwhelmed by them • Techniques to create emotional distance and develop constructive perspectives • Strategies to foster self-compassion and resilience
Relationships and Communication	• The protective effects of social relationships against stress and burnout • Understanding nonverbal communication in social interactions • Tips for effective communication and conflict resolution • Practical exercises to strengthen social skills
Problem-Solving and Coping Strategies	• The link between stress, worry, and problem-solving • Structured problem-solving techniques to address challenges effectively • Development of a personalized problem-solving plan
Positive Psychology	• Insights from happiness research, including sensory experiences and flow state • The role of gratitude in emotional well-being, with practical exercises to cultivate gratitude • Identifying personal strengths and finding ways to apply them
Applying Strategies in Daily Life	• Reflection on personal stress levels, energy, and optimism • Review of key topics and strategies for long-term well-being • Practical guidance on integrating learned techniques into everyday life

The program promotes active self-monitoring through brief daily and biweekly questionnaires on mood, exhaustion, and disengagement, each providing immediate, multimodal feedback and simple progress visualizations.

*reviga* is a purely internet-based web application accessible via standard web browsers on desktops, tablets, and smartphones; it requires no software installation and does not use cookies. The program adheres to the EU General Data Protection Regulation (GDPR) and German data protection laws. All user data is transmitted using TLS encryption and hosted exclusively in ISO 27001-certified data centers in Germany. All personal data are automatically deleted after program completion or a defined period of inactivity. More information on the program can be found in previous publications^25,26^.

### Measures

Baseline data, as well as follow-up data at 3 months (T1) and 6 months (T2) post-randomization, were collected using a secure and encrypted online survey service (*Easyfeedback*). All data were obtained through self-report. Participants received email invitations to complete the assessments, and those who did not respond were followed up with up to three reminders and phone calls to encourage participation. The primary time point for assessing effectiveness was T1, with T2 serving as an additional time point to assess the durability of the effects.

### Primary endpoint

The primary endpoint was the validated German version of the Perceived Stress Scale-10 item version (PSS-10), a patient-reported outcome measure (PROM)^27,28^ at T1 (3 months post-randomization).

### Secondary endpoints

Secondary outcomes included (1) anxiety symptoms, assessed with the Generalized Anxiety Disorder Assessment (GAD-7)^29,30^; (2) functional impairment, measured by the Work and Social Adjustment Scale (WSAS)^31,32^; (3) burnout, assessed with the Oldenburg Burnout Inventory (OLBI)^33^; (4) health-related quality of life, measured by the Assessment of Quality of Life-8 Dimensions (AQoL-8D)^34,35^; and (5) absenteeism, assessed by the number of sick days in the past 90 days.

### Demographic and clinical characteristics

Standard demographic variables were obtained at baseline including age, sex, education (highest degree), marital status, and employment status. In addition, information on clinical variables (e.g., number of sick days in the past 30 days, previous use of psychotherapy) and concomitant treatment (currently in psychotherapy, intake of psychotropic medication or antidepressants, use of alternative treatments etc.) were obtained. The Web Screening Questionnaire (WSQ) was used to assess comorbidities in the sample^36^.

### User engagement and satisfaction

To assess satisfaction with *reviga*, participants were asked to rate how likely they were to recommend *reviga* to a friend or colleague on a scale from 0 (not likely at all) to 10 (extremely likely). Based on this data, the Net Promoter Score (NPS) was calculated. Additionally, satisfaction was measured using the German version of the Client Satisfaction Questionnaire (CSQ-8)^37^. Usage of *reviga* was quantified by assessing the number of days participants logged into the program. This information was obtained from simple system records indicating whether a login occurred on a given day.

### Statistical analyses

All analyses were conducted using *R*, version 4.4.1^38^.

We conducted intent-to-treat (ITT) analyses as the primary analysis for all outcomes, including all participants in the intervention and control groups, regardless of their intervention usage. By contrast, per-protocol (PP) analyses included only intervention participants who used *reviga* on at least two different days. Intervention effects at T1 were assessed using ANCOVA, adjusting for baseline values. Treatment effects are reported as baseline-adjusted mean differences with 95% CIs on the original scale. Between-group effect sizes were calculated using Cohen’s *d* based on estimated marginal means from the ANCOVA model (estimated marginal means) with the *R* package *emmeans*^39^.

Missing data were imputed using multiple imputation based on baseline values and sociodemographic and clinical variables (age, sex, weekly work hours, psychotherapy at baseline, and antidepressant use). Specifically, we applied von Hippel’s bootstrapped maximum likelihood multiple imputation method^40^, which improves standard multiple imputation by incorporating an explicit bootstrap step to better approximate the sampling distribution of missing data. The imputation process was performed using the *bootImpute* and *mice* packages in *R*^41^. Missing data in the PP analyses were handled using the same multiple imputation approach as in the primary analysis.

As a sensitivity analysis, we applied the jump-to-reference (J2R) imputation method, a conservative approach that assumes intervention group participants who dropped out followed the trajectory of the control group, effectively treating dropout as a loss of any intervention benefits^42,43^. This approach is conservative because imputing less favorable outcomes for the intervention group tends to attenuate the estimated treatment effect, thus providing a stringent test of the intervention’s effectiveness. J2R was performed using the *R* package *bootImpute*.

ITT, J2R, and PP analyses were also repeated at T2 to evaluate the durability of effects. Additionally, a responder analysis classified participants as responders if they showed a ≥ 6-point improvement at T1 (corresponding to 15% of the scale range^44^), and proportions of responders were compared using χ² tests.

Throughout, a significance level of α = 0.05 (two-sided) was applied. To control for multiplicity, a gatekeeping strategy was pre-specified in the primary analysis, testing secondary endpoints in a predefined order, with each endpoint analyzed only if the previous one reached statistical significance^45^.

### Sample size

An a priori power analysis, based on an anticipated effect size of *d* = 0.37^20^, with a power of 0.80 at an α = 0.05 resulted in a required minimum sample size of N = 232, n = 116 per arm. Considering an expected drop-out rate of 20% of participants, the minimum sample size was set a priori at N = 290 (n = 145 per arm).

## Results

### Description of trial participants

Participants were recruited between September 2023 and November 2023 (follow-up period up to June 2024) via online advertisement. 936 people were screened for participation. Of these, 290 met all specified inclusion criteria and were randomized to the intervention (n = 147) and control group (n = 143). Participant characteristics at baseline are presented in Table 2. Additional information on what constituted treatment as usual can be found in Supplementary Table S1.

**Table 2:** Participant demographics and clinical characteristics at baseline

	Control	*reviga*	Total
	n = 143	n = 147	n = 290
Age (mean [SD])	43.7 (9.3)	43.2 (9.3)	43.5 (9.3)
Sex (n [%])			
male	13 (9.1)	27 (18.4)	40 (13.8)
female	130 (90.9)	120 (81.6)	250 (86.2)
intersexual	0 (0)	0 (0)	0 (0)
Marital status (n [%])			
unmarried	46 (32.2)	58 (39.5)	104 (35.9)
married/registered civil partnership	73 (51.0)	64 (43.5)	137 (47.2)
living separately	7 (4.9)	7 (4.8)	14 (4.8)
divorced/registered civil partnership annulled	14 (9.8)	18 (12.2)	32 (11.0)
widowed/registered civil partnership deceased	3 (2.1)	0 (0.0)	3 (1.0)
Education (n [%])			
Lower Secondary School Certificate (Hauptschulabschluss)	0 (0.0)	2 (1.4)	2 (0.7)
Intermediate Secondary School Certificate (Realschulabschluss)	9 (6.3)	12 (8.2)	21 (7.2)
University of Applied Sciences Entrance Qualification (Fachhochschulreife)	4 (2.8)	7 (4.8)	11 (3.8)
General University Entrance Qualification (Abitur)	13 (9.1)	6 (4.1)	19 (6.6)
completed vocational training	31 (21.7)	31 (21.1)	62 (21.4)
completed university studies	86 (60.1)	89 (60.5)	175 (60.3)
Employment (n [%])			
employed part-time	59 (41.3)	61 (41.5)	120 (41.4)
employed full-time	84 (58.7)	86 (58.5)	170 (58.6)
Work hours per week	34.7 (8.6)	35.9 (9.4)	35.3 (9.0)
Sick days in past 30 days (%)			
0 sick days	52 (36.4)	50 (34.0)	102 (35.2)
1–5 sick days	31 (21.7)	31 (21.1)	62 (21.4)
6–10 sick days	40 (28.0)	51 (34.7)	91 (31.4)
10+ sick days	20 (14.0)	15 (10.2)	35 (12.1)
WSQ positive screening (n [%]) (multiple answers possible)			
any depressive disorder	68 (47.6)	69 (46.9)	137 (47.2)
generalized anxiety disorder	131 (91.6)	132 (89.8)	263 (90.7)
panic disorder (without agoraphobia)	53 (37.1)	62 (42.2)	115 (39.7)
panic disorder with agoraphobia	20 (14.0)	20 (13.6)	40 (13.8)
agoraphobia (without panic disorder)	36 (25.2)	31 (21.1)	67 (23.1)
specific phobia	81 (56.6)	91 (61.9)	172 (59.3)
post-traumatic stress disorder	89 (62.2)	90 (61.2)	179 (61.7)
obsessive-compulsive disorder	43 (30.1)	44 (29.9)	87 (30.0)
alcohol use disorder	3 (2.1)	5 (3.4)	8 (2.8)
suicidality	0 (0)	0 (0)	0 (0)
Currently in psychotherapy (n [%])	31 (21.7)	39 (26.5)	70 (24.1)
Ever been in psychotherapy (n [%])	97 (67.8)	102 (69.4)	199 (68.6)
Currently taking any psychotropic medication^a^ (n [%])	23 (16.1)	24 (16.3)	47 (16.2)
Currently taking antidepressants^b^ (n [%])	18 (12.6)	17 (11.6)	35 (12.1)

^a^ATC classification codes N05/N06.

^b^ATC classification code N06A.

**Fig. 2:**
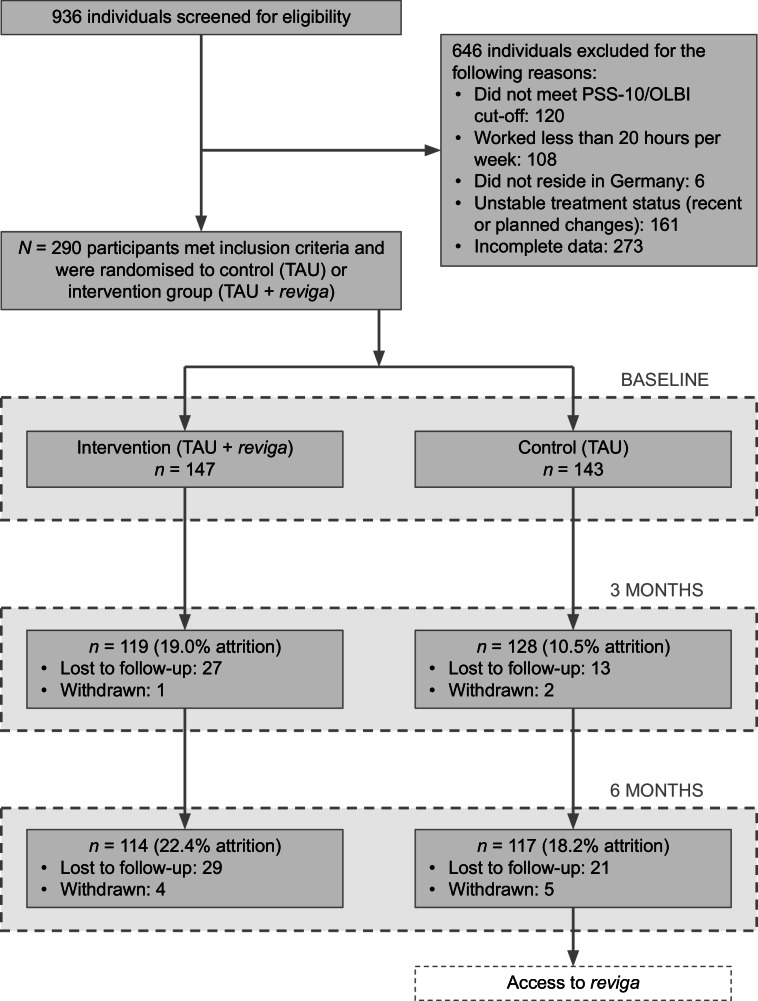
Participant flow chart. TAU treatment as usual.

Participants were in their mid-40s on average, mostly female (86.2%) and well-educated (60.3% university graduates). About 60% were employed full-time, while the rest worked part-time. Sick leave varied, with about a third reporting no days off in the past month, while a similar proportion had more than 10 days of sick leave. Mental health screening showed high rates of positive results for generalized anxiety (90.7%), post-traumatic stress disorder (61.7%), specific phobia (59.3%), and depression (47.2%). Importantly however, the WSQ overestimates the true prevalence of diagnoses and should not be interpreted as a clinical assessment^46^. Psychotherapy history was reported by about two-thirds of participants, and around one in ten were currently taking antidepressants. Reasons for exclusion, as well as attrition and reasons for drop-out are provided in the study flow chart (Fig. 2). Supplementary Table S2 provides a comparison of baseline characteristics between dropouts and completers. Supplementary Tables S3 and S4 contain information on changes in TAU between the different time points of assessment.

### Study outcomes

**Fig. 3:**
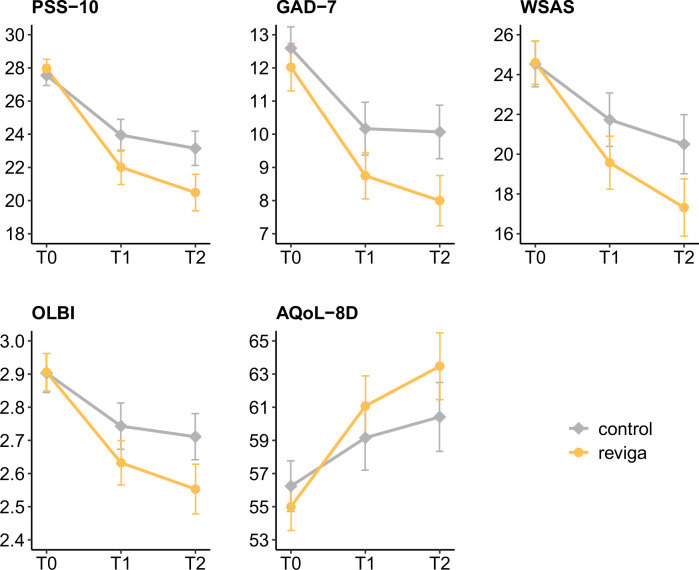
Mean scores for primary and secondary endpoints for the *reviga* group and control group across the study period, derived from the intention-to-treat (ITT) analysis. Error bars represent the 95% Confidence Interval (CI). Time points are T0 = Baseline, T1 = 3 months, T2 = 6 months. AQoL-8D Assessment of Quality of Life-8 Dimensions, GAD-7 Generalized Anxiety Disorder Assessment, OLBI Oldenburg Burnout Inventory, PSS-10 Perceived Stress Scale-10 items, WSAS Work and Social Adjustment Scale.

ITT analyses demonstrated significant intervention effects of small magnitude across all endpoints, except on absenteeism, at three and 6 months (see Table 3 and Fig. 3). After 3 months of using *reviga*, participants in the TAU + *reviga* intervention group reported a significantly larger reduction in perceived stress, the primary endpoint, than patients in the TAU-only control group: the estimated baseline-adjusted difference between the groups after 3 months was -2.1 points on the PSS-10 total score (95% CI = [−3.6, −0.7], *p* = 0.005, Cohen’s *d* = 0.36). A similar picture emerged for the secondary endpoints: After 3 months, participants in the intervention group reported significant reductions in anxiety (estimated baseline-adjusted group difference on the GAD-7 total score = −1.2 points, 95% CI = [−2.2, −0.1], *p* = 0.031; *d* = 0.28), difficulties regarding occupational and social functioning (estimated baseline-adjusted group difference on the WSAS total score = −2.2 points, 95% CI = [−4.1, −0.3], *p* = 0.024; *d* = 0.31) and burnout symptomatology (estimated baseline-adjusted group difference on the OLBI total score = −0.1 points, 95% CI = [−0.2, 0], *p* = 0.018; *d* = 0.31) when compared to the control group. In addition, health-related quality of life significantly improved in the intervention compared to the control group (estimated baseline-adjusted group difference on the AQoL-8D total score = 3.0 points, 95% CI = [0.7, 5.3], *p* = 0.01; *d* = 0.35). No significant between-group difference was found for absenteeism, quantified as the number of sick days (estimated baseline-adjusted group difference = 0.4 days, 95% CI = [−4.5, 5.2], *p* = 0.883; *d* = −0.02).

**Table 3:** Results of primary and secondary endpoints for ITT analyses

	Time	Control			*reviga*			ANCOVA			
		n	mean	SD	n	mean	SD	Treatment effect^a^ (95% CI)	*p*-value	Partial *η*^2^	Cohen’s *d* (95% CI)^b^
PSS-10	T0	143	27.6	3.7	147	28.0	3.5	-	-	-	-
	T1	143	23.9	5.8	147	22.0	6.4	−2.1 (−3.6, −0.7)	0.005	0.04	0.36 (0.12, 0.61)
	T2	143	23.2	6.3	147	20.5	6.8	−2.9 (−4.6, −1.2)	<0.001	0.05	0.46 (0.18, 0.74)
GAD-7	T0	143	12.6	3.9	147	12.0	4.4	-	-	-	-
	T1	143	10.2	4.9	147	8.7	4.3	−1.2 (−2.2, −0.1)	0.031	0.02	0.28 (0.02, 0.53)
	T2	143	10.1	4.9	147	8.0	4.7	−1.8 (−3, −0.7)	0.002	0.04	0.41 (0.15, 0.67)
WSAS	T0	143	24.5	7.0	147	24.6	6.8	-	-	-	-
	T1	143	21.7	8.2	147	19.6	8.2	−2.2 (−4.1, −0.3)	0.024	0.03	0.31 (0.04, 0.57)
	T2	143	20.5	9.1	147	17.3	8.9	−3.2 (−5.3, −1.1)	0.002	0.04	0.39 (0.14, 0.65)
OLBI	T0	143	2.9	0.4	147	2.9	0.3	-	-	-	-
	T1	143	2.7	0.4	147	2.6	0.4	−0.1 (−0.2, 0)	0.018	0.03	0.31 (0.06, 0.55)
	T2	143	2.7	0.4	147	2.6	0.5	−0.2 (−0.3, −0.1)	0.003	0.04	0.40 (0.13, 0.66)
AQoL-8D	T0	143	56.2	9.3	147	55.0	8.9	-	-	-	-
	T1	143	59.2	11.9	147	61.1	11.3	3.0 (0.7, 5.3)	0.01	0.03	0.35 (0.09, 0.62)
	T2	143	60.4	12.7	147	63.5	12.4	4.1 (1.6, 6.6)	0.002	0.05	0.42 (0.16, 0.68)
Sick days	T0	143	12.1	21.1	147	14.3	23.9	-	-	-	-
Sick days	T1	143	15.5	26.1	147	17.5	26.9	0.4 (−4.5, 5.2)	0.883	0	−0.02 (−0.26, 0.23)
	T2	143	13.0	24.5	147	12.4	23.8	−1.9 (−7, 3.2)	0.461	0.01	0.09 (−0.16, 0.35)

^a^Group difference on original scale 3 months (T1) and 6 months (T2) after baseline, adjusted for baseline scores.

^b^Based on baseline-adjusted means; positive values show effects in favor of the intervention group.

After 6 months, the ITT analyses showed sustained, and even increased, significant improvements for all endpoints, except for absenteeism. Perceived stress was significantly reduced in the intervention compared to the control group (estimated baseline adjusted group difference on the PSS-10 total score = −2.9 points, 95% CI = [−4.6, −1.2], *p* < 0.001; *d* = 0.46). Similarly, we found significantly larger reductions for anxiety (estimated baseline-adjusted group difference on the GAD-7 total score = -1.8 points, 95% CI = [−3.0, −0.7], *p* = 0.002; *d* = 0.41), difficulties in occupational and social functioning (estimated baseline-adjusted group difference on the WSAS total score = −3.2 points, 95% CI = [−5.3, −1.1], *p* = 0.002; *d* = 0.39) and burnout symptoms (estimated baseline-adjusted group difference on the OLBI total score = −0.2 points, 95% CI = [−0.3, −0.1], *p* = 0.003; *d* = 0.40) in the intervention compared to the control group. Larger improvements for health-related quality of life were also found for the AQoL-8D (estimated baseline-adjusted group difference on the AQoL-8D total score = 4.1 points, 95% CI = [1.6, 6.6], *p* = 0.002; *d* = 0.42) whereas the between-group difference in absenteeism remained negligible (estimated baseline-adjusted group difference = −1.9 days, 95% CI = [−7, 3.2], *p* = 0.461; *d* = 0.09).

Engagement with *reviga* was high, with 95.2% of participants in the intervention group (140 of 147) registering for use. On average, they interacted with the program on 17 different days (SD = 18.0), and 87.8% (129 of 147) met the predefined minimum usage threshold of at least 2 days. As a result, the PP dataset included 272 participants (129 from the intervention group and all 143 controls). PP analyses indicated slightly stronger intervention effects (see Table 4), and a conservative sensitivity analysis using J2R imputation consistently supported all findings (see Supplementary Table S5).

**Table 4:** Results of primary and secondary endpoints for PP analyses

	Time	Control			*reviga*			ANCOVA			
		n	mean	SD	n	mean	SD	Treatment effect^a^ (95% CI)	*p*-value	Partial *η*^2^	Cohen’s *d* (95% CI)^b^
PSS-10	T0	143	27.6	3.7	129	28.0	3.7	-	-	-	-
	T1	143	24.0	5.8	129	22.0	6.6	−2.2 (−3.8, −0.6)	0.006	0.04	0.38 (0.11, 0.65)
	T2	143	23.2	6.3	129	20.1	6.8	−3.4 (−5, −1.8)	<0.001	0.07	0.54 (0.27, 0.81)
GAD-7	T0	143	12.6	3.9	129	12.0	4.5	-	-	-	-
	T1	143	10.2	4.9	129	8.6	4.3	−1.3 (−2.5, −0.2)	0.017	0.03	0.32 (0.06, 0.59)
	T2	143	10.1	4.9	129	7.9	4.8	−1.9 (−3.1, −0.8)	0.001	0.05	0.43 (0.17, 0.70)
WSAS	T0	143	24.5	7.0	129	24.8	6.7	-	-	-	-
	T1	143	21.7	8.2	129	19.4	8.1	−2.4 (−4.3, −0.5)	0.011	0.03	0.34 (0.08, 0.60)
	T2	143	20.6	9.1	129	16.9	8.7	−3.8 (−5.9, −1.8)	<0.001	0.06	0.47 (0.22, 0.73)
OLBI	T0	143	2.9	0.4	129	2.9	0.3	-	-	-	-
	T1	143	2.7	0.4	129	2.6	0.4	−0.1 (−0.2, 0)	0.02	0.03	0.31 (0.05, 0.56)
	T2	143	2.7	0.4	129	2.5	0.5	−0.2 (−0.3, −0.1)	<0.001	0.05	0.46 (0.19, 0.72)
AQoL-8D	T0	143	56.2	9.3	129	54.6	9.0	-	-	-	-
	T1	143	59.2	11.9	129	61.3	11.3	3.5 (1.2, 5.7)	0.003	0.04	0.41 (0.14, 0.67)
	T2	143	60.4	12.7	129	63.8	12.3	4.8 (2.2, 7.5)	<0.001	0.06	0.50 (0.22, 0.79)
Sick days	T0	143	12.2	21.2	129	14.5	24.0	-	-	-	-
	T1	143	15.6	26.1	129	17.5	26.9	0.1 (−5.2, 5.4)	0.959	0.01	−0.01 (−0.27, 0.26)
	T2	143	13.1	24.7	129	12.8	24.3	−1.7 (−6.9, 3.5)	0.532	0.01	0.08 (−0.17, 0.33)

^a^Group difference on original scale 3 months (T1) and 6 months (T2) after baseline, adjusted for baseline scores.

^b^based on baseline-adjusted means; positive values show effects in favor of the intervention group.

Responder analysis based on complete cases further suggested a higher rate of clinically relevant improvements (defined as ≥6 points on the PSS-10) in the intervention group than in the control group, though the difference did not reach statistical significance. At T1, 42.9% (51 of 119) of intervention participants were classified as responders compared to 35.9% (46 of 128) in the control group (χ² = 1.24, *p* = 0.266; OR = 1.34, 95% CI [0.80, 2.23]).

### Adverse effects and user satisfaction

No adverse events related to the use of *reviga* or any adverse device effects were observed. The average score on the NPS was 6.7 (SD = 2.7) at T1 and 6.5 (SD = 3.0) at T2, suggesting that, overall, participants were more likely to recommend *reviga* to a friend or colleague than not. Mean score on the CSQ-8 was 19.8 (SD = 1.3) at T1 and 19.7 (SD = 1.7) at T2.

## Discussion

The LAVENDER-RCT aimed to assess the effectiveness of *reviga*, a self-guided digital intervention based on CBT among individuals experiencing work-related stress. The findings indicate that *reviga* leads to a significant reduction in stress-related symptoms and an improvement in functioning and health-related QoL after 3 months (T1), with these effects increasing in magnitude after 6 months (T2). No significant intervention effects were observed on sick days. Results were confirmed and remained consistent in a conservative sensitivity analysis, which assumed that any dropout in the intervention group would result in a loss of intervention effects. Pre-specified PP analyses suggested stronger intervention effects in participants who used *reviga* beyond initial exposure (i.e., on at least two different days). Given the prevalence of work-related stress and limited employer support, these findings highlight *reviga* as a scalable option for individuals with limited access to in-person resources.

With a standardized effect size of *d* = 0.36 on perceived stress after 3 months, *reviga* performs in line with the *d* = 0.37 average effect size reported in a meta-analysis of digital interventions for employee well-being^20^, supporting its expected effectiveness and reinforcing the validity of the results. Importantly, data suggest that *reviga* outperforms other CBT-based approaches, which show a lower average effect size of *d* = 0.25^20^. This suggests that *reviga* may offer a particularly effective application of relevant CBT principles in a digital format. A contributing factor could be its integration of key features identified as enhancing effectiveness and engagement for digital interventions for stress and burnout, such as persuasive system design features like tailoring and self-monitoring and secondary delivery modalities (e.g., email and text messages)^20^. Interestingly, *reviga* demonstrated increasing effects on all outcomes over time, with effect sizes on the primary endpoint, perceived stress, reaching *d* = 0.46 after 6 months. Work-related behaviors, including stress responses and coping mechanisms, are developed over long periods and may take time to unlearn, particularly when they have become habitual or reinforced by workplace dynamics. Thus, as users repeatedly engage with therapeutic content, refine their decision-making patterns, and apply newly acquired skills more confidently in their routines, improvements may become more pronounced over time. Such a trajectory aligns with theories of cognitive and behavioral adaptation, which propose that sustained engagement is necessary for new strategies to be ingrained and yield meaningful change^47^. Consistently, PP analyses revealed stronger effects (up to *d* = 0.54 for perceived stress at T2), suggesting that participants who engaged more actively with the intervention experienced greater benefits.

*reviga* had broad positive effects on the sequelae of chronic stress, including significant reductions in burnout and anxiety as well as improvements in functioning and health-related quality of life. However, it did not show an effect on sick days. This may be due to the fact that more than half of participants had minimal or no sick days at baseline, reducing the potential to detect an intervention effect. Absenteeism is also a lagging indicator, suggesting that longer follow-up may be needed to assess whether use of *reviga* may lead to fewer sick days in the long term.

Overall, the intervention effects observed for *reviga* are comparable with in-person occupational interventions in terms of impact on psychosocial outcomes^20^. At the same time, the improvements observed in the control group suggest TAU also contributed to stress reduction, potentially reflecting growing awareness and accessibility of mental health resources in the workplace in Germany^48^. Moreover, the experience of study participation itself, with its structured assessments, reflection prompts, and increased attention to stress-related symptoms, may have facilitated improvements^49^. While between-group differences at T1 were therefore modest, the increase in *reviga*’s effects over time suggests that it may provide additional long-term benefits beyond TAU as users become more adept in applying their knowledge and acquired skills in their work routines, as discussed above.

While the intervention effects of *reviga* are promising, there is potential to enhance user satisfaction, especially when compared to structurally similar interventions recently developed and evaluated by the same developer in other indications^21–23^. One possible explanation may be that individuals experiencing work-related stress and burnout often develop cognitive biases associated with heightened negativity^50,51^, which may have influenced their perception of the intervention. Thus, future research should examine ways to optimize *reviga* for different user profiles. Research suggests that human guidance in digital interventions may be an important factor to enhance engagement and effectiveness in general^52,53^ and in people with burnout specifically^20,54^; however, use of human resources must be carefully balanced with the need for scalability. As a complementary approach, personalization supported by large language models could help provide guidance in digital interventions within the boundaries of evidence-based and responsible implementation^55^. Determining the most effective way to implement such solutions remains an important task for future research.

The present RCT has several key strengths. First, with a total sample size of N = 290, this trial is among the largest conducted to date on digital interventions for work-related stress and burnout, significantly enhancing the validity and generalizability of its findings^20^. Second, the study adopted a pragmatic design, ensuring that it closely mirrors real-world conditions. Its broad inclusion and exclusion criteria capture the diverse experiences of individuals facing work-related stress and burnout, further strengthening the study’s relevance and impact. Third, the trial incorporated follow-up assessments at two time points (3 and 6 months post-randomization), allowing for a comprehensive evaluation of both immediate and sustained intervention effects.

We also acknowledge several limitations. First, participant dropout remains a relevant factor. While the present dropout rates are within an expected range and similar to those observed in previous trials of structurally comparable interventions^21–24^, understanding reasons for dropout, such as perceived burden, motivation, or user expectations, could provide insights for further optimizing the intervention. Second, while the results at T1 were statistically significant for the primary endpoint, they did not meet the pre-defined threshold for clinical relevance. In the absence of a validated minimally important clinical difference, we had to rely on a generic, distribution-based criterion to define response^44^. However, this may not be the most appropriate cut-off for identifying clinically meaningful change in perceived stress. Additionally, the primary endpoint assessed general rather than work-related stress, which may have been too unspecific to show clinically relevant changes after only 3 months. Third, our sample predominantly consisted of women. This overrepresentation may be partially explained by sex differences in the experience of burnout^56,57^. Additionally, women are more likely to seek help for mental health concerns, including utilizing psychotherapy and other specialized services^58,59^. This trend also extends to digital interventions, where a majority of users are women^60,61^. As such, further data could strengthen our understanding of *reviga*’s effectiveness for men. Fourth, digital interventions for stress and burnout show promise but may not be universally applicable. Individuals hesitant to engage with digital platforms may be beyond their reach and were likely underrepresented due to online recruitment. Consequently, findings may primarily generalize to those equipped for and motivated to use digital interventions rather than the broader population experiencing burnout that could benefit from CBT-based interventions^21^.

In summary, *reviga* demonstrates consistent effectiveness in reducing stress, anxiety, and burnout symptoms while improving functioning and health-related quality of life, with effects increasing over 6 months. Notably, as a fully self-guided intervention, *reviga* requires no additional human resources, making it a highly scalable solution. PP analyses further support its effectiveness, showing stronger effects among participants who engaged beyond initial exposure. These findings contribute to the growing evidence base supporting digital self-help interventions as an effective and accessible approach for managing work-related stress and burnout. Given the increasing demand for evidence-based digital mental health treatments and the limited availability of in-person stress management programs, *reviga* has the potential to address a critical gap in workplace mental health care.

## Supplementary information

Supplementary Information

## Data Availability

The datasets used and/or analyzed during the current study are not publicly available due to proprietary reasons but are available from the corresponding author on reasonable request.

## Code availability

The R code created/used during the current study is not publicly available due to proprietary reasons but is available from the corresponding author on reasonable request.
