# Supplementary material for: A randomized controlled trial of an interactive digital therapeutic for stress and burnout management
Source: Npj Ment Health Res. 2025 Dec 30;4:69. doi: 10.1038/s44184-025-00184-0 (PMC12753769; doi:10.1038/s44184-025-00184-0)
Supplement: Supplementary file 1 — Supplementary Information [file 44184_2025_184_MOESM1_ESM.pdf]

Supplementary Material for

"Effectiveness and safety of an interactive cognitive-behavioral digital intervention for stress and burnout: a randomized controlled trial"

Supplementary Table S1| Additional baseline variables characterizing "treatment as usual"

	**Control group**	***reviga***	**Total**
	n = 143	n = 147	n = 290
Number of doctor visits in past 3 months (mean [SD])	3.43 (4.51)	2.96 (3.92)	3.19 (4.22)
Number of hospital stays in past 3 months (mean [SD])	0.10 (0.31)	0.06 (0.27)	0.08 (0.29)
Curative stays in past 3 months (n [%])	5 (3.50)	4 (2.72)	9 (3.10)
Use of alternative therapies [1] (n [%])	35 (24.48)	29 (19.73)	64 (22.07)
Use of other online offers (e.g., apps) (n [%])	36 (25.17)	35 (23.81)	71 (24.48)

[1]Includes among others: acupuncture, dietary supplements, enzyme therapy, homeopathy, nutritional advice, relaxation, TMC

Supplementary Table S2| Comparison of baseline characteristics of dropouts and completers (up to T1)

	Dropout	Completer	Statistical Comparison
	n = 43	n = 247	
**Age (mean [SD])**	43.81 (8.21)	43.39 (9.48)	$t = 0.31$, $p = 0.761$
**Sex (n [%])**			$\chi^2 = 3.80$, $p = 0.051$
male	10 (23.3)	30 (12.1)	
female	33 (76.7)	217 (87.9)	
intersexual	0 (0)	0 (0)	
**Marital status (n [%])**			$\chi^2 = 12.02$, $p = 0.017$
unmarried	12 (27.9)	92 (37.2)	
married / registered civil partnership	17 (39.5)	120 (48.6)	
living separately	2 (4.7)	12 (4.9)	
divorced / registered civil partnership annulled	11 (25.6)	21 (8.5)	
widowed / registered civil partnership deceased	1 (2.3)	2 (0.8)	
**Education (n [%])**			$\chi^2 = 2.54$, $p = 0.770$
Lower Secondary School Certificate (Hauptschulabschluss)	1 (2.3)	1 (0.4)	
Intermediate Secondary School Certificate (Realschulabschluss)	4 (9.3)	17 (6.9)	
University of Applied Sciences Entrance Qualification (Fachhochschulreife)	2 (4.7)	9 (3.6)	
General University Entrance Qualification (Abitur)	3 (7.0)	16 (6.5)	

	Dropout	Completer	Statistical Comparison
completed vocational training	9 (20.9)	53 (21.5)	
completed university studies	24 (55.8)	151 (61.1)	
**Employment (n [%])**			$\chi^2 = 1.62$, $p = 0.203$
employed part-time	14 (32.6)	106 (42.9)	
employed full-time	29 (67.4)	141 (57.1)	
**Work hours per week**	37.77 (10.93)	34.90 (8.59)	$t = 1.64$, $p = 0.108$
**Sick days in past 30 days (%)**			$\chi^2 = 5.89$, $p = 0.117$
0 sick days	12 (27.9)	90 (36.4)	
1-5 sick days	6 (14.0)	56 (22.7)	
10+ sick days	16 (37.2)	75 (30.4)	
6-10 sick days	9 (20.9)	26 (10.5)	
**WSQ positive screening (n [%])** (multiple answers possible)			
any depressive disorder	20 (46.5)	117 (47.4)	$\chi^2 = 0.01$, $p = 0.917$
generalized anxiety disorder	39 (90.7)	224 (90.7)	$\chi^2 = 0.00$, $p = 1.00$
panic disorder (without agoraphobia)	22 (51.2)	93 (37.7)	$\chi^2 = 2.79$, $p = 0.095$
panic disorder with agoraphobia	9 (20.9)	31 (12.6)	$\chi^2 = 2.16$, $p = 0.141$
agoraphobia (without panic disorder)	11 (25.6)	56 (22.7)	$\chi^2 = 0.17$, $p = 0.676$
specific phobia	30 (69.8)	142 (57.5)	$\chi^2 = 2.29$, $p = 0.130$
post-traumatic stress disorder	31 (72.1)	148 (59.9)	$\chi^2 = 2.30$, $p = 0.130$
obsessive-compulsive disorder	17 (39.5)	70 (28.3)	$\chi^2 = 2.19$, $p = 0.139$

	Dropout	Completer	Statistical Comparison
alcohol use disorder	1 (2.3)	7 (2.8)	$\chi^2 = 0.00$, $p = 1.00$
suicidality	0 (0)	0 (0)	n/a
**Currently in psychotherapy (n [%])**	10 (23.3)	60 (24.3)	$\chi^2 = 0.02$, $p = 0.884$
**Ever been in psychotherapy (n [%])**	29 (67.4)	170 (68.8)	$\chi^2 = 0.03$, $p = 0.857$
**Currently taking any psychotropic medication[a] (n [%])**	9 (20.9)	38 (15.4)	$\chi^2 = 0.83$, $p = 0.362$
**Currently taking antidepressants[b] (n [%])**	6 (14.0)	29 (11.7)	$\chi^2 = 0.17$, $p = 0.681$
**PSS-10**	28.09 (3.83)	27.70 (3.63)	$t = 0.62$, $p = 0.539$
**GAD-7**	13.05 (4.33)	12.17 (4.19)	$t = 1.23$, $p = 0.224$
**WSAS**	23.60 (7.26)	24.74 (6.83)	$t = -0.96$, $p = 0.342$
**OLBI**	2.94 (0.37)	2.90 (0.35)	$t = 0.72$, $p = 0.477$
**AQoL-8D**	54.20 (9.76)	55.86 (9.02)	$t = -1.04$, $p = 0.301$

[a] ATC classification codes N05 / N06
[b] ATC classification code N06A

Supplementary Table S3| Start of treatment between the different time points of assessment (T0, T1, T2)

	**Control group**	***reviga***
**Start of treatment between T0 and T1**		
Antidepressants (n [%])	1/143 (0.7)	4/147 (2.72)
Psychotherapy (n [%])	9/130 (6.92)	9/130 (6.92)
Treatment in general [a] (n [%])	10/130 (7.69)	13/130 (10.0)
**Start of treatment between T0 and T2**		
Antidepressants (n [%])	5/143 (3.50)	6/147 (4.08)
Psychotherapy (n [%])	13/119 (10.92)	10/124 (8.06)
Treatment in general [a] (n [%])	17/119 (14.29)	16/124 (12.9)

[a] Either receiving psychotherapy or taking antidepressants

Supplementary Table S4| End of treatment between the different time points of assessment (T0, T1, T2)

	**Control group**	***reviga***
**End of treatment between T0 and T1**		
Antidepressants (n [%])	4/143 (2.80)	8/147 (5.44)
Psychotherapy (n [%])	6/142 (4.23)	8/138 (5.80)
Treatment in general [a] (n [%])	10/142 (7.04)	16/141 (11.35)
**End of treatment between T0 and T2**		
Antidepressants (n [%])	3/143 (2.10)	8/147 (5.44)
Psychotherapy (n [%])	8/141 (5.67)	2/138 (1.45)
Treatment in general [a] (n [%])	11/141 (7.8)	10/142 (7.04)

[a] Either receiving psychotherapy or taking antidepressants

Supplementary Table S5| Results of primary and secondary endpoints for J2R analyses

	**Time**	**Control**			***reviga***			**ANCOVA**			
		n	mean	SD	n	mean	SD	Treatment effect[a] (95% CI)	*p*-value	Partial η^2	Cohen's *d* (95% CI)[b]
PSS-10	T0	143	27.6	3.7	147	28.0	3.6	-	-	-	-
	T1	143	24.0	5.8	147	22.5	6.4	-1.8 (-3, -0.5)	0.006	0.03	0.31 (0.09, 0.52)
	T2	143	23.3	6.4	147	21.1	6.8	-2.4 (-3.7, -1.2)	< .001	0.04	0.39 (0.19, 0.59)
GAD-7	T0	143	12.6	3.9	147	12.0	4.5	-	-	-	-
	T1	143	10.2	4.9	147	9.0	4.5	-1.0 (-1.8, -0.2)	0.02	0.02	0.23 (0.04, 0.42)
	T2	143	10.1	4.9	147	8.4	4.8	-1.4 (-2.2, -0.6)	0.001	0.03	0.32 (0.13, 0.5)
WSAS	T0	143	24.6	6.9	147	24.6	6.8	-	-	-	-
	T1	143	21.8	8.2	147	19.9	8.2	-1.9 (-3.4, -0.4)	0.013	0.02	0.26 (0.06, 0.47)
	T2	143	20.7	9.0	147	18.0	9.1	-2.8 (-4.4, -1.2)	< .001	0.03	0.34 (0.15, 0.54)
OLBI	T0	143	2.9	0.4	147	2.9	0.3	-	-	-	-
	T1	143	2.7	0.4	147	2.7	0.4	-0.1 (-0.2, 0)	0.013	0.02	0.26 (0.06, 0.46)
	T2	143	2.7	0.4	147	2.6	0.5	-0.1 (-0.2, 0)	0.005	0.02	0.28 (0.09, 0.47)
AQoL-8D	T0	143	56.2	9.3	147	55.0	8.9	-	-	-	-
	T1	143	59.0	12.1	147	60.5	11.4	2.6 (0.8, 4.4)	0.005	0.03	0.31 (0.09, 0.52)
	T2	143	60.0	12.9	147	62.1	12.6	3.3 (1.4, 5.2)	< .001	0.03	0.35 (0.16, 0.54)
Sick days	T0	143	12.3	21.3	147	14.3	23.9	-	-	-	-
	T1	143	15.8	26.2	147	17.7	27.0	0.2 (-3.8, 4.3)	0.906	0	-0.18 (-0.33, -0.04)
	T2	143	13.5	24.5	147	13.0	24.3	-1.6 (-5.8, 2.5)	0.435	0	-0.09 (-0.21, 0.03)

[a] Group difference on original scale 3 months (T1) and 6 months (T2) after baseline, adjusted for baseline scores.

[b] based on baseline-adjusted means; positive values show effects in favor of the intervention group.

CONSORT 2010 checklist of information to include when reporting a randomised trial*

Section/Topic	Item No	Checklist item	Reported on page No
**Title and abstract**			
	1a	Identification as a randomised trial in the title	1
	1b	Structured summary of trial design, methods, results, and conclusions (for specific guidance see CONSORT for abstracts)	2
**Introduction**			
Background and objectives	2a	Scientific background and explanation of rationale	3-4
	2b	Specific objectives or hypotheses	4
**Methods**			
Trial design	3a	Description of trial design (such as parallel, factorial) including allocation ratio	5-9
	3b	Important changes to methods after trial commencement (such as eligibility criteria), with reasons	n/a
Participants	4a	Eligibility criteria for participants	6
	4b	Settings and locations where the data were collected	5
Interventions	5	The interventions for each group with sufficient details to allow replication, including how and when they were actually administered	6-7 + Table 1 + Fig. 1
Outcomes	6a	Completely defined pre-specified primary and secondary outcome measures, including how and when they were assessed	7-8
	6b	Any changes to trial outcomes after the trial commenced, with reasons	n/a
Sample size	7a	How sample size was determined	9
	7b	When applicable, explanation of any interim analyses and stopping guidelines	n/a
Randomisation:			
Sequence generation	8a	Method used to generate the random allocation sequence	5
	8b	Type of randomisation; details of any restriction (such as blocking and block size)	5
Allocation concealment mechanism	9	Mechanism used to implement the random allocation sequence (such as sequentially numbered containers), describing any steps taken to conceal the sequence until interventions were assigned	5
Implementation	10	Who generated the random allocation sequence, who enrolled participants, and who assigned participants to interventions	5

Blinding	11a	If done, who was blinded after assignment to interventions (for example, participants, care providers, those assessing outcomes) and how	n/a
	11b	If relevant, description of the similarity of interventions	n/a
Statistical methods	12a	Statistical methods used to compare groups for primary and secondary outcomes	8-9
	12b	Methods for additional analyses, such as subgroup analyses and adjusted analyses	8-9
**Results**			
Participant flow (a diagram is strongly recommended)	13a	For each group, the numbers of participants who were randomly assigned, received intended treatment, and were analysed for the primary outcome	Fig. 2
	13b	For each group, losses and exclusions after randomisation, together with reasons	Fig. 2
Recruitment	14a	Dates defining the periods of recruitment and follow-up	9
	14b	Why the trial ended or was stopped	n/a
Baseline data	15	A table showing baseline demographic and clinical characteristics for each group	Table 2
Numbers analysed	16	For each group, number of participants (denominator) included in each analysis and whether the analysis was by original assigned groups	Tables 3-4
Outcomes and estimation	17a	For each primary and secondary outcome, results for each group, and the estimated effect size and its precision (such as 95% confidence interval)	Tables 3-4
	17b	For binary outcomes, presentation of both absolute and relative effect sizes is recommended	
Ancillary analyses	18	Results of any other analyses performed, including subgroup analyses and adjusted analyses, distinguishing pre-specified from exploratory	Table 4 + Supp. Materials
Harms	19	All important harms or unintended effects in each group (for specific guidance see CONSORT for harms)	11
**Discussion**			
Limitations	20	Trial limitations, addressing sources of potential bias, imprecision, and, if relevant, multiplicity of analyses	12-15
Generalisability	21	Generalisability (external validity, applicability) of the trial findings	12-15
Interpretation	22	Interpretation consistent with results, balancing benefits and harms, and considering other relevant evidence	12-15
**Other information**			
Registration	23	Registration number and name of trial registry	5
Protocol	24	Where the full trial protocol can be accessed, if available	5
Funding	25	Sources of funding and other support (such as supply of drugs), role of funders	16

Citation: Schulz KF, Altman DG, Moher D, for the CONSORT Group. CONSORT 2010 Statement: updated guidelines for reporting parallel group randomised trials. BMC Medicine. 2010;8:18.
© 2010 Schulz et al. This is an Open Access article distributed under the terms of the Creative Commons Attribution License (http://creativecommons.org/licenses/by/2.0), which permits unrestricted use, distribution, and reproduction in any medium, provided the original work is properly cited.

*We strongly recommend reading this statement in conjunction with the CONSORT 2010 Explanation and Elaboration for important clarifications on all the items. If relevant, we also recommend reading CONSORT extensions for cluster randomised trials, non-inferiority and equivalence trials, non-pharmacological treatments, herbal interventions, and pragmatic trials. Additional extensions are forthcoming: for those and for up-to-date references relevant to this checklist, see www.consort-statement.org.
